# Supplementary material for: Relationship between the composition of the intestinal microbiota and the tracheal and intestinal colonization by opportunistic pathogens in intensive care patients
Source: PLoS One. 2020 Aug 28;15(8):e0237260. doi: 10.1371/journal.pone.0237260 (PMC7454957; doi:10.1371/journal.pone.0237260)

**SUPPLEMENTARY MATERIAL**

**Relationship between the composition of the intestinal microbiota and the tracheal and intestinal colonization by opportunistic pathogens in intensive care patients.**

Candice Fontaine, Laurence Armand-Lefèvre, Mélanie Magnan, Anissa Nazimoudine, Jean-François Timsit, Etienne Ruppé

**Supplementary Figure 1**: Intestinal and endotracheal relative abundances of MDR-GNB in feces (n=18) and in endotracheal aspirations (EA, n=9). The difference was significant (Student test, p=0.03).


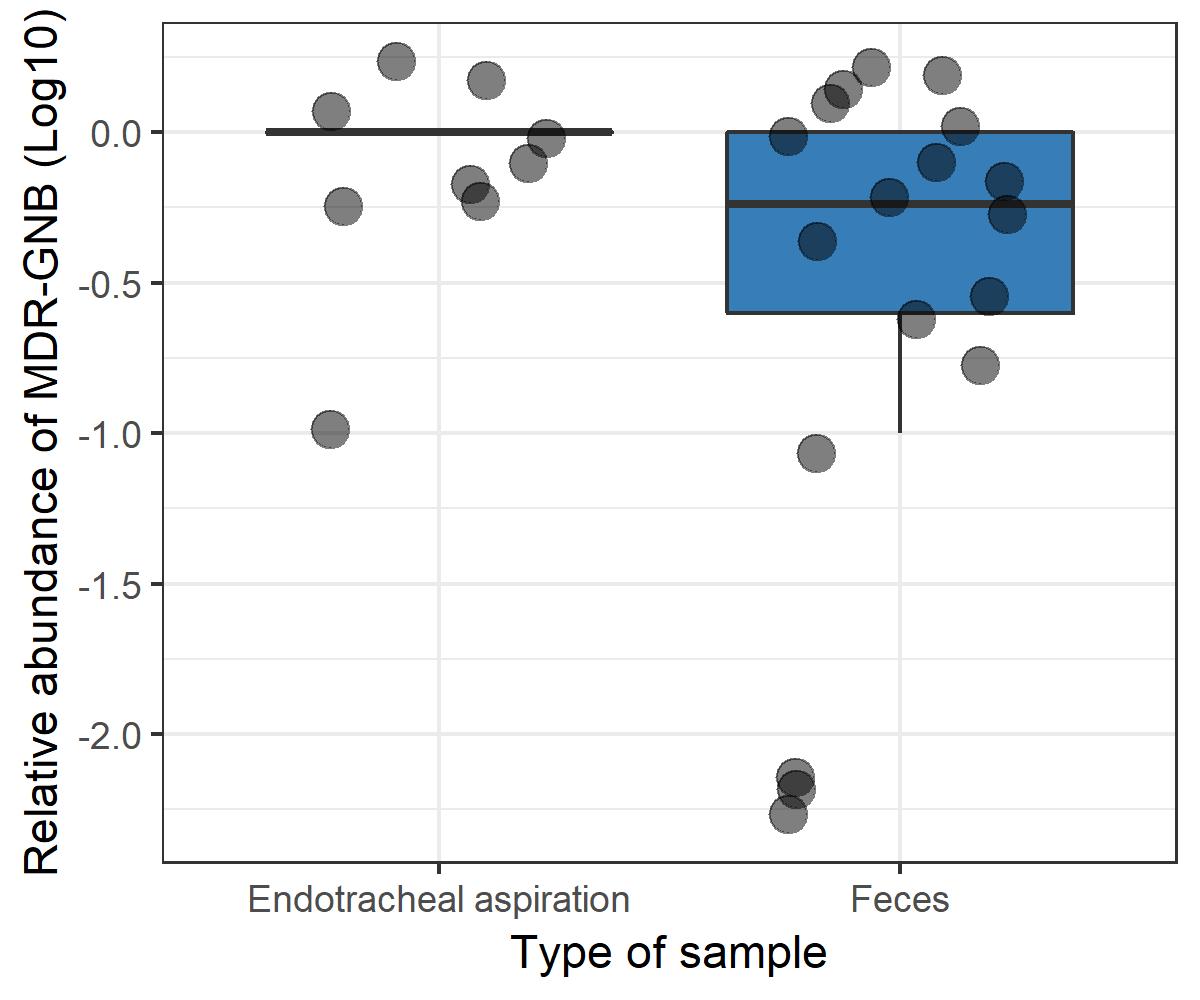


**Supplementary Figure 2:** *Enterococcus* spp. and *Enterococcus faecium* intestinal colonization according to the composition of the intestinal microbiota with one sample considered per patient (the first feces passed after admission, n=24 samples with 16S profiling data). Panels A and B: boxplot superimposed by dot-plot of Shannon diversity index (A) and richness (B) according to the detection by culture of *E. faecium*. Panels C and D: Dot-plot of the relative abundance of reads assigned to *Enterococcus* spp. (in Log10), Shannon diversity index (C) and richness (D). The shaded grey area depicts the 95% confidence interval around the black line. Panels A and B: Student test; panels C and D: Pearson correlation test.


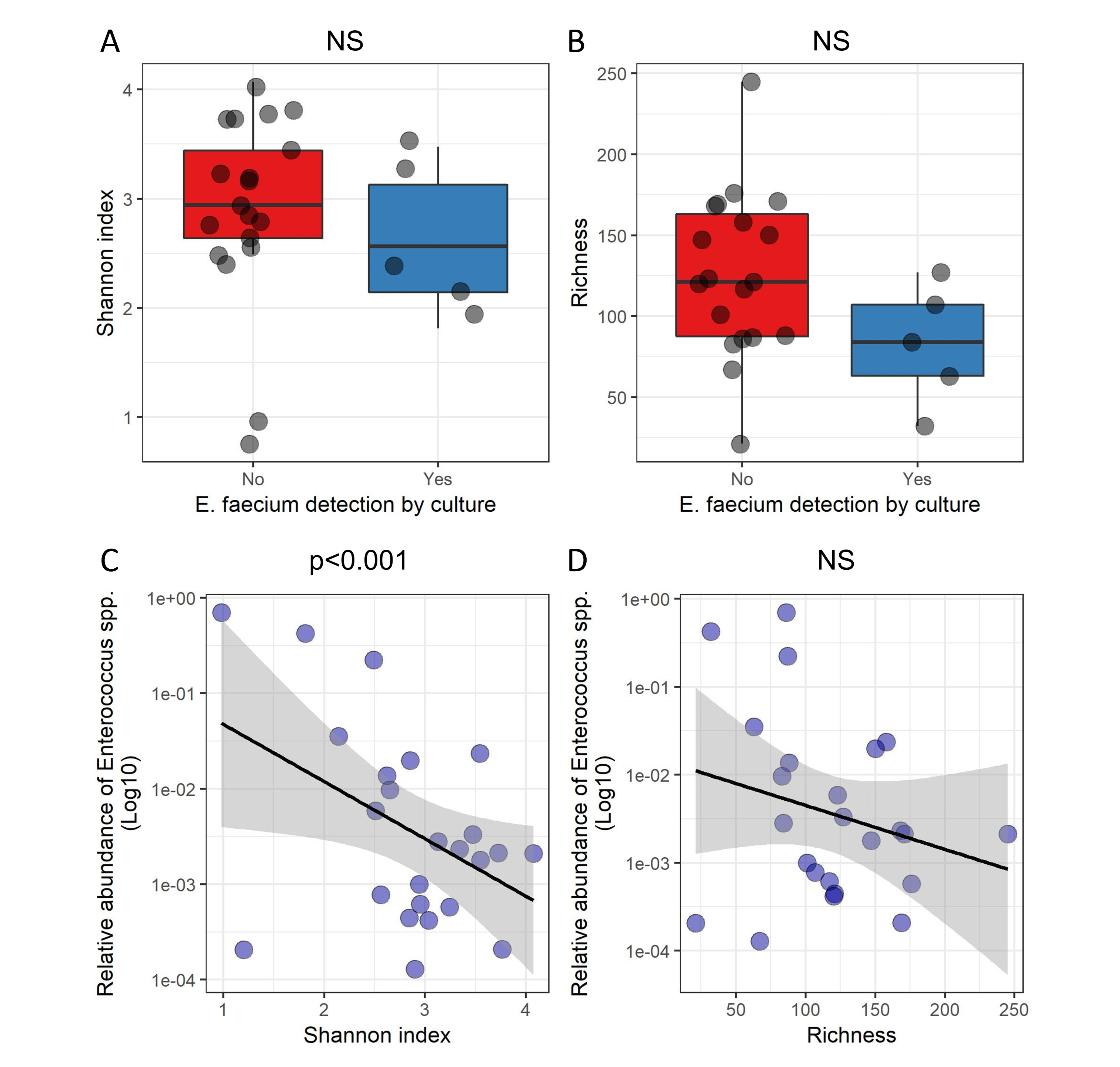


**Supplementary Figure 3: Yeast detection in culture according to the composition of** the intestinal microbiota (n=48 samples). Panels A and B: boxplot superimposed by dot-plot of Shannon diversity index (A) and richness (B) according to the detection by culture of yeasts. The Student t test was used (p=NS for both panels).


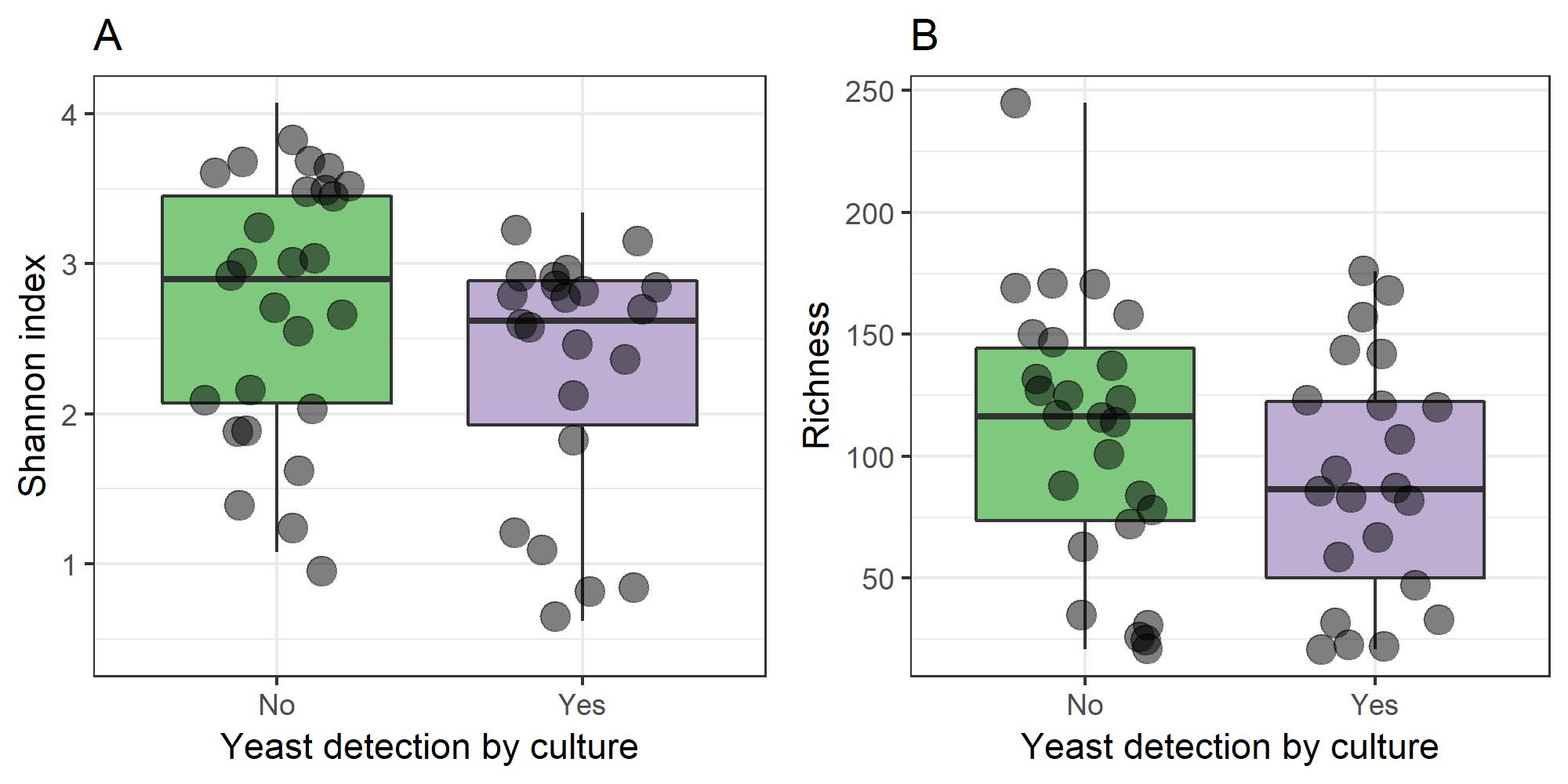


**Supplementary Figure 4:** Intestinal concentration (panel A) and relative abundance (panel B) of multidrug-resistant Gram-negative Bacilli (MDR-GNB) and their presence in endotracheal aspiration (EA) (n=18 samples). Student tests were performed.


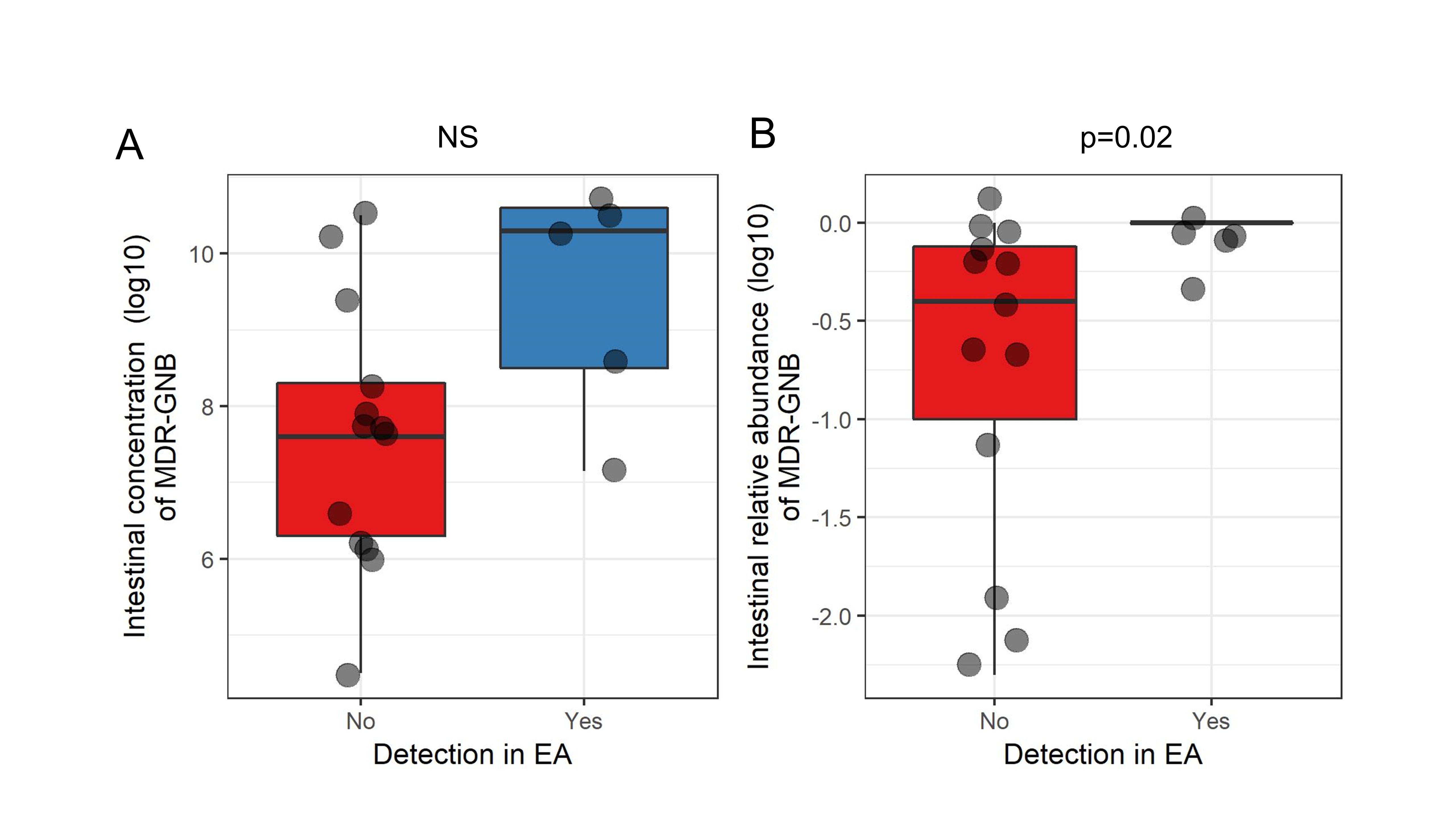

Supplement: S1 File — (DOCX) [file pone.0237260.s001.docx]
